# NKG2D Signaling Leads to NK Cell Mediated Lysis of Childhood AML

**DOI:** 10.1155/2015/473175

**Published:** 2015-07-08

**Authors:** Patrick Schlegel, Kerstin Ditthard, Peter Lang, Markus Mezger, Sebastian Michaelis, Rupert Handgretinger, Matthias Pfeiffer

**Affiliations:** Department of Hematology and Oncology, University Children's Hospital Tübingen, University of Tübingen, Hoppe-Seyler-Straße 1, 72076 Tübingen, Germany

## Abstract

Natural killer cells have been shown to be relevant in the recognition and lysis of acute myeloid leukemia. In childhood acute lymphoblastic leukemia, it was shown that HLA I expression and KIR receptor-ligand mismatch significantly impact ALL cytolysis. We characterized 14 different primary childhood AML blasts by flow cytometry including NKG2D ligands. Further HLA I typing of blasts was performed and HLA I on the AML blasts was quantified. In two healthy volunteer NK cell donors HLA I typing and KIR genotyping were done. Blasts with high NKG2D ligand expression had significantly higher lysis by isolated NK cells. Grouping the blasts by NKG2D ligand expression led to a significant inverse correlation of HLA I expression and cytolysis in NKG2D low blasts. Furthermore, a significant positive correlation of NKG2D ligand expression and blast cytolysis was shown. No impact of KIR ligand-ligand mismatch was found but a significantly increased lysis of homozygous C2 blasts by KIR2DL1 negative NK cells (donor B) was revealed. In conclusion, NKG2D signaling leads to NK cell mediated lysis of childhood AML despite high HLA I expression.

## 1. Introduction

Despite intensification of therapy and the use of new chemotherapeutic agents, one-third to one-half of children with acute myeloid leukemia (AML) experience relapse [1, 2]. Hematopoietic stem cell transplantation (HSCT) and natural killer (NK) cell transfer as cellular targeted treatment strategy have been shown to increase relapse-free survival in childhood AML [3]. Natural killer cells are cytotoxic lymphocytes that play an important role in antitumor immunity [4]. Reduced NK cell count, impaired NK cell function, and the prognostic relevance in leukemia evidence the involvement of NK cells in leukemia immunosurveillance [5–7]. Moreover, evidence for the ability of NK cells to recognize and eliminate leukemic blasts in humans has been provided by clinical HSCT trials [8, 9]. NK cells are regulated by activating, inhibitory and co-receptor signaling. The activation comprises the principles of “missing self” and “induced self,” implying that NK cells kill target cells with low or absent expression of HLA class I and stress-induced expression of ligands for activating NK cell receptors as well as costimulatory receptors [10]. In pediatric ALL, susceptibility to NK cell mediated recognition and cytolysis is correlated to the quantity of HLA I expression and KIR receptor-ligand (RL) mismatch [11, 12]. However, little is known about childhood AML in regard to NK cell mediated antitumor effects including quantity of HLA I expression as well as the prostimulatory signaling of DNAM-1 and NKG2D. In addition, whereas the relevance of NKG2D signaling in NK cell immunosurveillance and escape mechanism of adult AML is well established, its role in childhood AML is unknown [13]. To address the question of which activating and inhibitory signals determine NK cell mediated recognition and cytolysis in childhood AML, we analyzed primary childhood AML blasts and their susceptibility to NK cell mediated cytolysis in a HLA mismatched setting, taking into account major features of NK cell regulation.

## 2. Materials and Methods

The study was authorized by the ethical institutional review board of the University of Tübingen, Germany. The blasts used in the experiments were isolated from patients who were treated at the Department of Pediatric Hematology/Oncology of the University Children's Hospital Tübingen, Germany. Patients and healthy donors gave informed consent.

### 2.1. HLA I Typing of Cryopreserved Childhood AML Blasts and Healthy Donors

HLA I typing of the selected cryopreserved AML blasts was provided by the institute for transplant immunology and immunohematology (see Table 1).

### 2.2. KIR Genotyping of Healthy Donors

*Donor A, KIR A haplotype* was KIR2DL1, KIR2DL3, KIR2DL4, KIR2DS4, KIR3DL1, KIR3DL2, and KIR3DL3.* Donor B, KIR B haplotype* was KIR2DL2, KIR2DS2, KIR2DL4, KIR2DS4, KIR3DL1, KIR3DS1, KIR3DL2, and KIR3DL3.

KIR genotype of donor A resulted in B0 score and KIR genotype of donor B resulted in B3 score (http://www.ebi.ac.uk/cgi-bin/ipd/kir/donor_b_content.cgi) [14]. Amplification of KIR genes was performed using KAPA Sybr Fast qPCR Master Mix for iCycler (PEQLAB, Erlangen, Germany). After an initial denaturation step for 20 s at 95°C, 32 PCR cycles with 3 s at 95°C and 20 s at 64°C were run on the CFX96 real-time PCR detection (Bio-Rad, Hercules, CA, USA) system as published [15].

### 2.3. Preparation of CD56^+^CD3^−^ NK Effector Cells

Peripheral blood mononuclear cells (PBMCs) were isolated from peripheral whole blood of two healthy volunteer donors by density gradient centrifugation using Biocoll separating solution (Biochrom GmbH, Berlin, Germany). CD56^+^CD3^−^ NK cells were isolated from PBMCs by immunomagnetic CD56^+^ selection using microbeads (Miltenyi Biotech, Bergisch Gladbach, Germany), followed by CD3^+^ depletion using dynabeads (Invitrogen, Carlsbad, CA, USA) [16].

### 2.4. Leukemic Blasts

Acute myeloid leukemia cells were obtained from pediatric patients from bone marrow or peripheral blood at the time of diagnosis or relapse after informed consent of the legal guardians. Diagnoses were childhood AML (FAB classification M0, M2, M4, M5, M5b, and M6). AML blasts were cryopreserved immediately after diagnosis (purity > 80%). The relative proportion of the primary childhood AML blasts was reliably determined by flow cytometry, using an extensive immunophenotyping leukemia panel including the markers CD45, CD33, CD34, CD117, HLADR, AC133, MPO, CD15, CD13, CD7, CD17, Glycophorin A, CD56, CD1a, CD3, CD4, CD8, CD5, CD64, w65, CD41a, CD14, CD15, CD61, CD2, CD42, CD79b, CD19, CD10, CD20, CD22, Kappa, Lambda, TdT, and W6/32. AML blast samples below 80% purity of blasts were considered ineligible and excluded.

### 2.5. Phenotypic Characterization of Childhood AML Blasts

Flow cytometry was performed according to standard protocols on a 4-color FACSCalibur flow cytometer using CellQuest software for data acquisition (Becton Dickinson, Heidelberg, Germany). AML blasts were not distinguished from healthy leukocytes by any marker but purity of blasts > 80% was a prerequisite. The listed antibodies were used in saturating concentrations: mouse anti-human antibodies CD11a (IgG_2a_, PE), CD18 (IgG_1_, FITC), CD48 (IgM, FITC), CD50 (IgG_1_, PE), CD54 (IgG_2b_, PE), CD58 (IgG_2a_, PE), CD95 (IgG_1_, FITC), and CD112 (IgG_1_, FITC) (Becton Dickinson); MICA and MICB (IgG_2a_, APC) and HLA-ABC (IgG_2a_, unlabeled) (Biolegend, San Diego, CA, USA); CD155 (IgG_1_, FITC) (eBioscience, San Diego, CA, USA); unlabeled ULBP1 (Z-9, IgG_2a_), ULBP2 (E16, IgG_2a_), ULBP3 (2F9, IgG_2a_), and ULBP4 (6E6, IgG_2b_) (Santa Cruz, Dallas, TX, USA); HLA-E (IgG_1_, unlabeled) (Exbio, Praha, Czech Republic); polyclonal goat anti-mouse (Gt F(ab′)_2_, FITC) (Dako, Hamburg, Germany); polyclonal goat anti-mouse (Gt F(ab′)_2_, PE) (Becton Dickinson). Quantitative analyses of HLA class I and HLA-E expression were done according to manufacturer's instructions (Qifikit, Dako). One antigen binding site was assumed per one antigen molecule. Mean Fluorescence Intensity Ratios (MFIR) were calculated by dividing the mean fluorescence signals from AML blasts by the corresponding isotype control. A ratio > 2 was defined positive and a ratio > 10 was defined highly positive. Analyses of flow cytometric data were performed using FCS Express 4.0 (De Novo Software, Glendale, CA, USA).

### 2.6. Cytotoxicity Assay

Cytolytic activity of NK cells was measured in a 2 h BATDA [bis(acetoxymethyl)2,2:6,2-terpyridine-6,6-dicarboxylate] europium release assay. Cryopreserved primary childhood AML blasts and the erythroblastoid cell line K562 (M6 leukemia) were used as target cells. No enrichment of primary childhood AML blasts was done: purity > 80% blasts. K562 was used as positive control to exclude functional NK cell inactivity. Target cells (leukemic blasts) were labeled with 3 *μ*L of the fluorescence enhancing ligand BATDA (Perkin Elmer, Waltham, MA, USA) for 60 min at 37°C. After five washing steps, the target cell suspension was adjusted to 2 × 10^5^ cells/well and seeded into microplates (5000 cells/well). Four different effector to target (E : T) ratios were tested with and without IL2 preincubation overnight (Proleukin, Basel, Switzerland). The assays were done as published [16]. Specific lysis was calculated as follows: %-specific lysis = (experimental release − spontaneous release)/(maximum release − spontaneous release)  *∗*  100.

### 2.7. Statistical Analysis

Analysis was done using GraphPad Prism Version 5.04. *p* values < 0.05 were considered significant. Unpaired and paired *t*-tests as well as Pearson's correlation coefficient were used. For the comparison of donor A and donor B, the pairing considered the blasts (e.g., blast number 1–blast number 1); the comparing condition was the NK activity (specific lysis).

## 3. Results

### 3.1. HLA I and HLA-E Expression on Childhood AML Blasts

**Figure 1:**
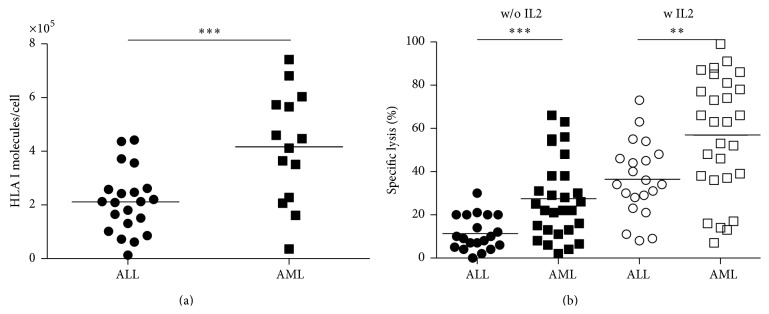
Quantitative HLA I expression (a) in primary childhood ALL and AML blasts measured by Qifikit (Dako). HLA I molecules/cell mean 211064 ± 25800 (range: 13149 to 436661) in ALL and 416339 ± 55329 in AML (range: 35724 to 741641). AML showed significantly higher HLA I expression than ALL (*n*_AML_ = 14, *n*_ALL_ = 21; *p* = 0.0007). (b) Cellular cytotoxicity (BATDA europium release assay, Perkin Elmer) of freshly isolated CD56^+^ enriched and CD3^+^ depleted NK cells from healthy volunteer donors towards ALL (only 1 donor, *n*_ALL_ = 21) versus AML (2 different donors, *n*_AML_ = 14) blasts with and without IL2 prestimulation at E : T ratio 10 : 1 revealed significantly higher lysis in AML than ALL without IL2 prestimulation (filled symbols) and with IL2 prestimulation (blank symbols) (*p*_w/o  IL2_ = 0.0007; *p*_IL2_ = 0.0038). Comparing ALL versus AML lysis in single donor comparison also revealed significantly higher lysis for AML blasts. The ALL data was already published in Br J Haematology 2007 [11].

The mean HLA I expression was 416339 ± 55329 molecules per AML blast (range: 35724 to 741642/cell) (Figure 1(a)). AML blasts showed higher expression of HLA class I compared to pediatric ALL blasts. The ALL data was already published in Br J Haematology 2007 [11]. In contrast, HLA-E expression in childhood AML blasts was low (range: 0 to 2700 molecules/cell). Comparing NK cell mediated cytotoxicity without and with IL2 prestimulation revealed a higher susceptibility of AML blasts, despite higher mean expression of HLA I molecules per blast (Figure 1(b)).

### 3.2. Impact of HLA I Expression and KIR Ligand Mismatch on the Lysis of AML Blasts

**Figure 2:**
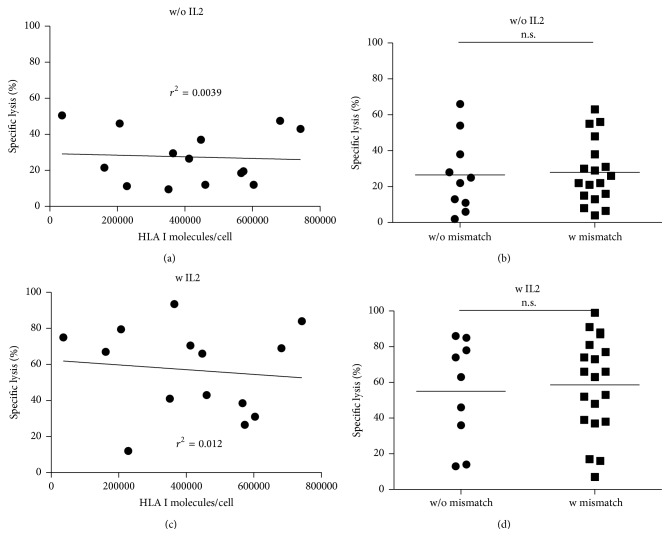
(a) Quantitative HLA I expression measured by Qifikit (Dako) was correlated to the mean NK cell mediated lysis (2 donors) of primary childhood AML blasts at E : T ratio 10 : 1. NK cells were obtained from two healthy volunteer donors. PBMCs were isolated from peripheral whole blood by density gradient centrifugation using Biocoll separating solution (Biochrom) followed by a two-step immunomagnetic CD56^+^ enrichment and subsequent CD3^+^ depletion. No correlation was found without IL2 prestimulation (*n*_AML_ = 14; Pearson *r* = −0.06, *r* ^2^ = 0.0039, *p* = n.s.). (b) Distribution of blast HLA type was C1/C1 (*n* = 5), C1/C2 (*n* = 5), and C2/C2 (*n* = 4). KIR ligand-ligand match versus mismatch did not reveal any difference in specific lysis of primary childhood AML blasts (*n*_w/o  mismatch_ = 10, *n*_mismatch_ = 18;  *p* = 0.84). Donor A revealed a KIR ligand-ligand match versus mismatch proportion (3 to 11) and donor B revealed a KIR ligand-ligand match versus mismatch proportion (7 to 7). (c) No correlation was found with IL2 prestimulation at E : T ratio 10 : 1 (*n*_AML_ = 14; Pearson *r* = −0.11, *r* ^2^ = 0.012, *p* = n.s.). (d) There was no impact of KIR ligand-ligand match or mismatch on IL2 prestimulated NK cell mediated cytolysis of AML blasts.

There was no correlation of HLA I expression and NK cell mediated cytolysis of childhood AML blasts independent from E : T ratio and without or with IL2 prestimulation (*n* = 14, donors A and B, E : T 10 : 1, without IL2, Pearson *r* = −0.06, *p* = 0.83; with IL2, *r* = −0.11, *p* = 0.70) (Figures 2(a) and 2(c)). Furthermore, no correlation was found in single donor analysis. HLA-E expression was not correlated with blast lysis in donor A or B in any condition (Figure  S1 in Supplementary Material available online at http://dx.doi.org/10.1155/2015/473175).

The analysis of KIR ligand-ligand match versus mismatch revealed a proportion of (3 to 11) in donor A and (7 to 7) in donor B with difference neither without nor with IL2 prestimulation (Figures 2(b) and 2(d)). Furthermore, no correlation was found in single donor analysis. Further analysis by subdividing into a KIR ligand match and mismatch group did not result in a correlation of specific lysis and quantitative HLA I expression in any group.

### 3.3. NK Cell Cytolysis of Childhood AML Blasts and K562 Cell Line

Mean lysis of K562 blasts at E : T ratio 10 : 1 in donors A and B without IL2 prestimulation was 67.2 ± 6.0% and 73.2 ± 4.0% and with IL2 prestimulation was 82.1 ± 4.5% and 81.8 ± 4.7%, respectively. There was no significant difference comparing the lysis ability of donors A and B in any E : T ratio or by integrating the E : T ratios 20 : 1, 10 : 1, 5 : 1, and 2.5 : 1 without and with IL2 prestimulation and there was no difference comparing the increase of lysis by IL2 (Figures  S2 A, B, and C). IL2 significantly increased the lysis of K562 in both donors (*n*_A_ = 9, *p*_A_ < 0.0001; *n*_B_ = 12, *p*_B_ = 0.0006) (Figures  S2 D and E). No differences in susceptibility to NK cell mediated lysis depending on FAB classification were observed.

**Figure 3:**
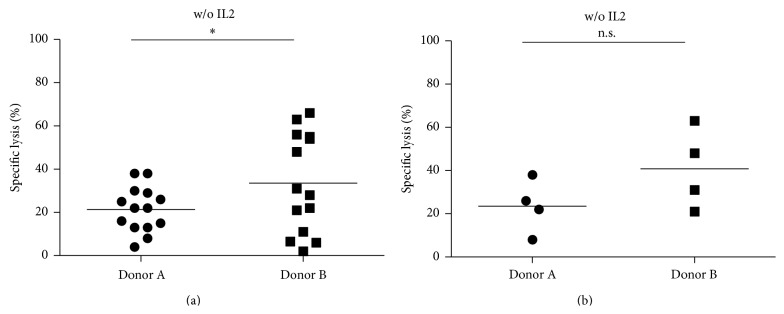
(a) Cytolysis of isolated NK cells comparing donor A (B content score 0) versus donor B (B content score 3) towards 14 primary childhood AML blasts at E : T ratio 10 : 1. PBMCs were isolated from peripheral whole blood from two healthy volunteer donors A and B by density gradient centrifugation using Biocoll separating solution (Biochrom) followed by a two-step immunomagnetic CD56^+^ enrichment and subsequent CD3^+^ depletion. Donor B showed significantly higher lysis than donor A (*n*_AML_ = 14, *p* = 0.038). (b) The analysis of blast lysis of blasts homozygous for C2 showed by trend a higher lysis of donor B (*n*_C2/2_ = 4, *p* = 0.06) applying the E : T ratio 10 : 1. Donor A was KIR2DL1 positive and donor B was KIR2DL1 negative. KIR2DL1 is the corresponding KIR receptor for the C2 HLA group.

In the tested childhood AML blasts, donor B showed significantly higher lysis than donor A (*n* = 14, all ETs, *p* = 0.0029) (Figure 3(a)). At an E : T ratio of 10 : 1, mean lysis was significantly higher in donor B than in donor A without IL2 prestimulation (21.4 ± 2.8% versus 33.5 ± 6.1%, *n* = 14, *p* = 0.038) but not with IL2 prestimulation (56.1 ± 6.1% versus 57.6 ± 8.5%). The increase of lysis by IL2 was significant in both donors (*n* = 14, *p*_A_ < 0.0001, *p*_B_ = 0.0002) but was significantly higher in donor A versus donor B (*n* = 14, all ETs, *p* = 0.0035). There was no influence of KIR ligand mismatch between NK donor and AML blasts on specific lysis within each donor. The activating KIR2DS2 and KIR3DS1 encoded by donor B were not found to significantly influence NK mediated cytolysis in this setting of these experiments. Yet, donor B showed a higher lysis of C2 homozygous AML blasts than donor A without and with IL2 prestimulation. The comparison at E : T 10 : 1 was not significant (*n*_C2/2_ = 4, *p*_w/o  IL2_ = 0.06) (Figure 3(b)) but was significantly higher comparing all ETs (*n*_C2/2_ = 4, all ETs, *p*_w/o  IL2_ = 0.008, *p*_w  IL2_ = 0.04). No difference was found for C1 homozygous AML blasts (*n*_C1/1_ = 5, *p*_w/o  IL2_ = 0.29, *p*_w  IL2_ = 0.102) and C1/C2 heterozygous blasts (*n*_C1/2_ = 4, *p*_w/o  IL2_ = 0.13, *p*_w  IL2_ = 0.83) without and with IL2 prestimulation (Figure  S3). The impact of the KIR ligand-ligand model published by the Perugia group (Velardi) in 2002 [8] did not reveal any differences in cytolysis of C1/C1, C1/C2, and C2/C2 blasts for C1/C2 donor A and C1/C1 donor B. The analyses included comparisons at all effector to target ratios (20 : 1, 10 : 1, 5 : 1, and 2.5 : 1) and pooled ET ratios (all 4 ETs).

### 3.4. Flow Cytometric Characterization of AML Blasts

**Figure 4:**
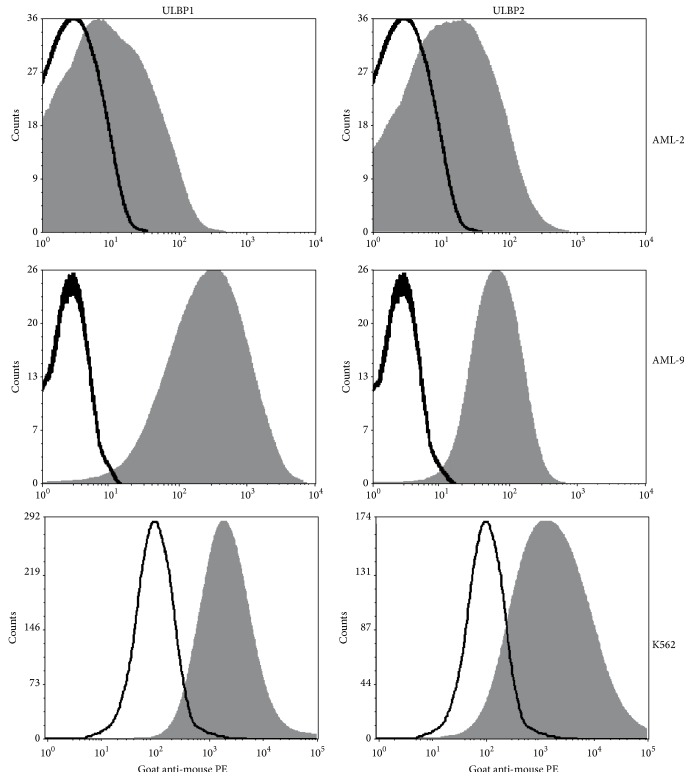
NKG2D ligand expression of the primary childhood AML blasts, AML-2 and AML-9, and the erythroblastoid cell line K562. Cells were incubated with unlabeled mouse anti-human ULBP1 and ULBP2 antibody and incubated in a second step with a PE labelled secondary goat anti-mouse antibody and measured on a FACSCalibur flow cytometer. Isotype control corresponds to the black line; the ULBP1 and ULBP2 positivity of the cells is displayed in shaded grey. In AML-2, 51.34% of the cells were ULBP1 positive and 49.40% were ULBP2 positive. In AML-9, 95.74% of the cells were ULBP1 positive and 95.12% were ULBP2 positive. In K562, 91.34% were ULBP1 positive and 86.14% were ULBP2 positive. The percentage of positive ULBP1 and ULBP2 cells was obtained by histogram subtraction method. The Median Fluorescence Intensity Ratio (MFIR) for AML-2 was 2.82 for ULBP1 and 3.34 for ULBP2; for AML-9, it was 117.67 for ULBP1 and 28.35 for ULBP2; for K562, it was 19.28 for ULBP1 and 15.35 for ULBP2. MFIR was calculated by the MFI goat anti-mouse PE divided by the MFI isotype control.

NKG2D ligands ULBP1-4 were heterogeneously expressed on the tested AML blasts (Figure 4). The total mean MRFI of ULBP1-4 was 25.8 ± 11.5 ranging from negative to highly positive (MRFI = 154, Table  S1). MICA and MICB were not detectable on the AML blasts. DNAM-1 ligand CD155 was low expressed but detectable on all blasts (MRFI ≥ 2); CD112 was highly expressed in most AML blasts (MRFI ≥ 10). The same pattern was found for LFA-1 (CD11a and CD18) as well as ICAM-3 (CD50) and LFA-3 (CD58). Heterogeneously expressed were ICAM-1 (CD54), SLAMF2 (CD48), and the FAS receptor CD95 (Table  S2).

### 3.5. NKG2D Ligand Expression and NK Cell Cytolysis

**Figure 5:**
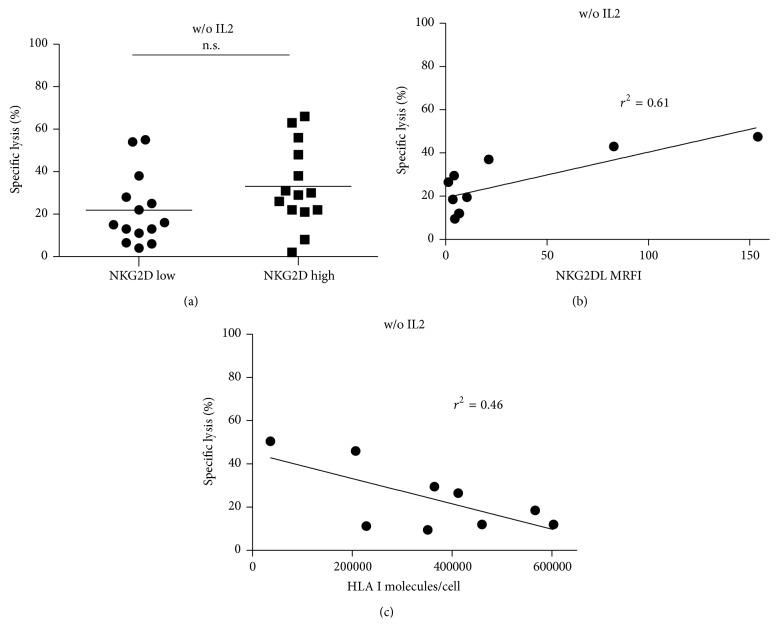
NKG2DL and HLA I expression importantly influence NK cell activation. (a) NKG2DL expression was measured semiquantitatively by calculating the MFIR (Mean Fluorescence Intensity Ratio). The blasts were grouped by the mean MFIR of ULBP1-4 into NKG2DL_low_ and NKG2DL^high^ blasts (mean MFIR 6.6; NKG2DL_low_ < 6.6, NKG2DL^high^ > 6.6). NKG2DL^high^ blasts were lysed by trend higher than NKG2DL_low_ blasts including the E : T ratio 10 : 1 of two healthy volunteer donors by immunomagnetic CD56^+^ enriched and CD3^+^ depleted NK cells (*n*_high_ = 7, *n*_low_ = 7; *p* = 0.11). (b) The blasts were grouped by HLA I expression. HLA I^high^ blasts were defined by HLA I molecules per cell within the range of the mean and standard deviation (SD) or higher; the HLA I_low_ blasts were below the mean minus SD. The NKG2DL expression significantly correlated to NK cell mediated cytolysis of two healthy volunteer donors in the HLA I^high^ blast group at E : T ratio 10 : 1 (*n* ^HLA-high^ = 10, Pearson *r* = 0.78; *p* = 0.0076). (c) NKG2DL_low_ blasts showed a significant inverse correlation of HLA I molecule expression per cell and NK cell mediated cytolysis of two healthy volunteer donors at E : T ratio 10 : 1 (*n*_NKG2D-low_ = 9, Pearson *r* = −0.68; *p* = 0.0426).

Grouping the blasts by the mean MRFI of ULBP1-4 expression (MRFI = 6.6) resulted in two groups with 7 blasts each. The lysis of the NKG2DL^high^ group was higher than cytolysis of the NKG2DL_low_ group without IL2 but was not significant at E : T 10 : 1, *p* = 0.11 (Figure 5(a)). Comparing all effector to target ratios at once showed significantly higher lysis of the NKG2DL^high^ group (*n* = 14, all ETs, *p* = 0.0111). With IL2 prestimulation, no difference between the cytolysis of NKG2DL^high^ and NKG2DL_low_ (*n* = 14, all ETs, *p* = 0.52) was found. Furthermore, we grouped the blasts into HLA I_low_ and HLA I^high^. Blasts with HLA I molecules higher than or within the range of the mean minus standard deviation were defined as HLA I^high^ blasts, whereas blasts with HLA I below the mean expression minus standard deviation were defined as HLA I_low_ blasts. Analyzing the HLA I^high^ blasts only, NK cytolysis showed significant positive correlation with NKG2D ligand expression (Pearson *r* = 0.78, *p* = 0.0076) (Figure 5(b)). Obversely excluding NKG2DL^high^ blasts with an absolute MRFI >10 cytolytic activity inversely correlated with HLA I expression (Pearson *r* = −0.68, *p* = 0.04) (Figure 5(c)).

## 4. Discussion

Improvement in the treatment of childhood AML has led to an overall survival of 60% [1]. Failure of treatment comprises relapse in 30–40% of patients and treatment related mortality. Relapsed patients face a clearly reduced overall survival of 16 to 42% [2] and are often in need for an allogeneic HSCT [17]. In HSCT, NK cells have been identified to play a crucial role in relapse-free survival [14]. In particular alloreactive GVL reactions have been demonstrated to reduce relapse rates in adult AML and pediatric ALL after HSCT [8, 15, 18, 19]. Three original different models have been established to predict NK alloreactivity starting with the ligand-ligand mismatch defined by the Perugia group if the donor has a HLA ligand that is absent in the recipient [8, 9], the Memphis model defined by the incompatibility of donor KIR receptor and recipient HLA ligand [20], and the Stanford KIR haplotype model defined by presence of activating KIR receptors [21]. For pediatric ALL, several factors have been described that influence NK susceptibility of ALL blasts like quantitative HLA class I expression and KIR receptor ligand mismatch between NK cells and blasts in graft versus host direction [11, 12] or expression of DNAM-1 ligands [22].

Here, we aimed at investigating* in vitro* the factors which may influence the NK mediated cytolysis of primary childhood AML blasts in a HLA mismatched model. We found that ALL and AML showed heterogeneous expression of HLA I with significantly higher expression (twice as high) in AML than ALL (Figure 1(a)). The ALL data was already published in Br J Haematology 2007 [11]. Despite this observation, NK cell cytolysis of AML was significantly higher than in tested ALL blasts (Figure 1(b)) indicating that proactivating factors such as DNAM-1 ligands and NKG2D ligands are involved in the cytolysis of AML blasts [23, 24].

In our tested pediatric AML blasts, the capability of lysing the blasts was increased in donor B (Figure 3(a)). Multidimensional differences included HLA type and KIR genotype. In the condition with IL2 prestimulation, there was no difference between donor A and donor B (Figure  S4 A). This might be a hint that cytokine activated NK cells, irrespective of the donor, can overcome inhibition of target cells by shifting the balance of regulation towards NK cell activation [25]. Whereas there was no difference in cytolysis regarding KIR ligand-ligand match versus mismatch and KIR RL match/mismatch regarding all blasts at once, subgrouping into blast HLA genotype C1 group and blast HLA genotype C2 group revealed increased lysis of donor B in blasts homozygous for HLA C2 in comparison to donor A without and with IL2 prestimulation (Figure 3(b), Figures  S4 and S5 B) [21]. Donor B lacked KIR2DL1 (KIR2DL1 gene not encoded), which is the corresponding KIR receptor for HLA C2. Thus homozygous C2 blasts express an inhibitory HLA I ligand for which donor B does not have a corresponding KIR receptor and cannot be inhibited hereby. A similar observation has been shown to be clinically relevant in T cell depleted haploidentical HSCT. The risk of relapse in ALL and AML was significantly determined by the reconstitution of NK KIR receptors [26].

In consequence, there must be other factors leading to sufficient AML blast lysis by NK cells [23, 25].* In vivo *and also proven* in vitro*, AML blasts hamper NK cell function by various mechanisms including T_reg_ induction, shedding of soluble NKG2D ligands (MIC and ULBP molecules), and direct cellular interaction inducing unfavorable KIR phenotype and reduction of proactivating NK cell receptors [13]. For instance, the high expression of DNAM-1 ligands CD112 and CD155 induces the downregulation of DNAM-1 NK coreceptor by receptor-ligand crosslinking [27]. Moreover, natural cytotoxicity receptors (NCRs) play a crucial role in the recognition and elimination of AML. The NCR^dull^ immunophenotype, most likely induced by cellular interaction of AML blasts and NK cells, is reversible during absence of AML blasts in complete remission and is associated with poor outcome, demonstrating the plasticity of NK cell receptor landscape and function by the close interaction with AML [28].

Two well-characterized and strong mechanisms of NK cell activation are NKG2D- and DNAM-1 signaling [29]. In ALL, blasts do express low density of NKG2D ligands compared to AML and CLL [30]. In pediatric ALL, blasts show significant higher NKG2DL expression than adult ALL blasts [22]. There is only little data published on pediatric AML. In contrast to childhood ALL blasts (own data not shown), childhood AML blasts heterogeneously expressed NKG2D ligands, ranging from low to very high levels of ULBP1-4 expression, with all blasts being negative for MICA and MICB (Figure 4 and Table  S1). This confirms and extends the finding in adult AML, at least a proportion of AML blasts being negative for MICA and MICB also in childhood AML [24, 31]. In our tested blasts, 64% were at least low positive for any NKG2D ligand and 28% were highly positive facilitating grouping the blasts into NKG2DL_low_ and NKG2DL^high^ phenotypes. DNAM-1 ligands were homogeneously expressed (Table  S2) and thus were not suitable to subgroup the blasts and to explain the different lysis of the tested AML blasts, even though DNAM-1 triggering is important in the lysis of several tumor targets including AML [23, 32]. By subgrouping the blasts according to NKG2DL expression, a difference of cytolysis was revealed (Figure 5(a) and Figure  S6), strongly indicating a major impact of NKG2D on NK cell mediated lysis of childhood AML blasts [24, 25, 30]. This hypothesis was further supported by a significant inverse correlation of HLA I expression and NK cell mediated cytolysis of NKG2DL_low_ blasts (Figure 5(c)). Conversely, a significant positive correlation was found for NKG2DL expression within the HLA I^high^ blasts (Figure 5(b)). In conclusion, both well-established prostimulatory factors of NK cell activation were confirmed: HLA I reduction and NKG2DL positivity determined NK cell mediated lysis of childhood AML blasts. Moreover, NKG2DL high expression was strong enough to override NK cell inhibition by HLA I expression. Obversely, blast 14 showed extremely low HLA I expression and was lysed very well despite lacking NKG2DL expression.

These results imply that, for immunotherapeutical approaches recruiting NK cells, a characterization of target cells including HLA I typing and HLA I quantification and characterization of surface markers on the blasts might help estimate how sensitive the targets might be for NK cells. Furthermore, characterization of the donor with regard to HLA typing, KIR genotyping, and KIR RL mismatch can possibly improve donor selection of NK cell immunotherapy or HSCT. According to the minimized* in vitro* cytolytic differences between donor A and donor B after cytokine stimulation with IL2 and the significant increase in cytolytic activity* ex vivo*, NK cell transfer in lymphodepletion after chemotherapy or* in vivo *NK cell stimulation by subcutaneous low-dose and long-term IL2 application should be considered as treatment options in AML patients [3, 33, 34]. To counteract escape mechanism of AML like downregulation of NKG2D ligands [13], NKG2D receptor induction in NK cells by* ex vivo* expansion [35, 36] or* in vivo* expansion plus activation by applied cytokines [34, 37], and on the other hand induction of NKG2D ligands on AML target cells by all-trans retinoic acid, the histone deacetylase inhibitor sodium valproate or spironolactone might increase clinically relevant NK mediated antitumor effect [38–41]. In conclusion, the inhibition of NK cells in childhood AML through high HLA I expression can be overridden by prostimulatory NKG2D signaling. The increased knowledge on childhood AML recognition and cytolysis by NK cells will aid in designing novel NK cell-targeting and optimizing immunotherapy approaches for the treatment of AML.

## Supplementary Material

The supplementary material provides additional analyses regarding the impact of HLA E on NK cell mediated cytolysis, comparative analyses of donor A and B taking into account blast HLA I genotype and NKG2DL high versus low expression on NK cytolysis.

## Figures and Tables

**Table 1:** 

Healthy donors	HLA type	HLA I ligand		Corresponding inhibitory KIR	KIR mismatch
Donor A	C1/C2	bw6^+^/bw4^+^	cw3^+^/cw4^+^	KIR2DL1, KIR2DL2/3, KIR3DL1	—
Donor B	C1/C1	bw6^+^/bw4^+^	cw3^+^/cw3^+^	KIR2DL2/3, KIR3DL1	KIR2DL1

Childhood AML blasts					
AML-1 FAB M0	C1/C1	bw6^+^/bw4^+^	**cw3** ^+^ **/cw3** ^+^	KIR2DL2/3, KIR3DL1	KIR2DL1
AML-2 FAB M0	C2/C2	bw6^+^/bw4^+^	cw4^+^/cw4^+^	KIR2DL1, KIR3DL1	KIR2DL2/3
AML-3 FAB M2	C1/C1	bw4^+^/bw4^+^	**cw3** ^+^ **/cw3** ^+^	KIR2DL2/3, KIR3DL1	KIR2DL1
AML-4 FAB M2	C1/C2	**bw6** ^+^ **/bw6** ^+^	cw3^+^/cw4^+^	KIR2DL1, KIR2DL2/3	KIR3DL1
AML-5 FAB M4	C2/C2	bw6^+^/bw4^+^	cw4^+^/cw4^+^	KIR2DL1, KIR3DL1	KIR2DL2/3
AML-6 FAB M4	C1/C2	bw6^+^/bw4^+^	cw3^+^/cw4^+^	KIR2DL1, KIR2DL2/3, KIR3DL1	—
AML-7 FAB M5	C2/C2	**bw6** ^+^ **/bw6** ^+^	cw4^+^/cw4^+^	KIR2DL1	KIR2DL2/3, KIR3DL1
AML-8 FAB M5	C1/C2	bw4^+^/bw4^+^	cw3^+^/cw4^+^	KIR2DL1, KIR2DL2/3, KIR3DL1	—
AML-9 FAB M5	C1/C1	bw6^+^/bw4^+^	**cw3** ^+^ **/cw3** ^+^	KIR2DL2/3, KIR3DL1	KIR2DL1
AML-10 FAB M5	C1/C1	**bw6** ^+^ **/bw6** ^+^	**cw3** ^+^ **/cw3** ^+^	KIR2DL2/3	KIR2DL1, KIR3DL1
AML-11 FAB M5b	C1/C2	bw6^+^/bw4^+^	cw3^+^/cw4^+^	KIR2DL1, KIR2DL2/3, KIR3DL1	—
AML-12 FAB M6	C1/C2	**bw6** ^+^ **/bw6** ^+^	cw3^+^/cw4^+^	KIR2DL1, KIR2DL2/3	KIR3DL1
AML-13 FAB M6	C1/C1	bw6^+^/bw4^+^	**cw3** ^+^ **/cw3** ^+^	KIR2DL2/3, KIR3DL1	KIR2DL1
AML-14 FAB M6	C2/C2	bw6^+^/bw4^+^	cw4^+^/cw4^+^	KIR2DL1, KIR3DL1	KIR2DL2/3
