# Supplementary material for: NKG2D Signaling Leads to NK Cell Mediated Lysis of Childhood AML
Source: J Immunol Res. 2015 Jul 8;2015:473175. doi: 10.1155/2015/473175 (PMC4510257; doi:10.1155/2015/473175)
Supplement: Supplementary file 1 — The supplementary material provides additional analyses regarding the impact of HLA E on NK cell mediated cytolysis, comparative analyses of donor A and B taking into account blast HLA I genotype and NKG2DL high versus low expression on NK cytolysis. [file 473175.f1.zip › Table_S1-1389920.docx]

**Table S1. Median Fluorescence Intensity Ratio of NKG2D ligands**

| **AML-Blast** | **FAB** | **ULBP1** | **ULBP2** | **ULBP3** | **ULBP4** |
| --- | --- | --- | --- | --- | --- |
| **AML-1** | M0 | 0.70 | 0.96 | 1.17 | 0.69 |
| **AML-2** | M0 | 2.82 | 3.34 | 3.16 | 1.17 |
| **AML-3** | M2 | 1,12 | 2,17 | 1,90 | 1,22 |
| **AML-4** | M2 | 0.48 | 0.93 | 0.54 | 0.53 |
| **AML-5** | M4 | 6.00 | 14.04 | 1.15 | *NA* |
| **AML-6** | M4 | 1.00 | 1.10 | 1.34 | 1.04 |
| **AML-7** | M5 | 7.55 | 21.30 | 5.02 | 3.40 |
| **AML-8** | M5 | 0.53 | 0.47 | 0.32 | *NA* |
| **AML-9** | M5 | 117.67 | 28.35 | 5.87 | 2.05 |
| **AML-10** | M5 | 60.67 | 10.67 | 9.68 | 1.85 |
| **AML-11** | M5b | 1.80 | 2.21 | 0.35 | 0.28 |
| **AML-12** | M6 | 0.98 | 1.03 | 1.40 | 0.72 |
| **AML-13** | M6 | 0.62 | 4.52 | 0.98 | 0.59 |
| **AML-14** | M6 | 0.86 | 2.57 | 2.77 | 0.89 |

ULBP1-4 Median Fluorescence Intensity Ratios (MFIR) of NKG2D ligands were calculated by MFI of goat anti-mouse PE divided by MFI of isotype control. To detect fluorescence, cells were incubated with unlabeled Mouse anti-Human ULBP1-4 antibody, then incubated with secondary PE labeled Goat anti-Mouse antibody and measured on a FACSCalibur^TM^ cytometer. AML-1 to AML-14 represents 14 primary childhood AML blasts. AML was classified according to the French American British Classification (FAB). MFIR < 2 was defined negative, MFIR ≥ 2 defined positive and MFIR ≥ 10 was defined highly positive. Results in AML-5 and AML-8 for ULBP4 were not applicable.
