# Supplementary material for: NKG2D Signaling Leads to NK Cell Mediated Lysis of Childhood AML
Source: J Immunol Res. 2015 Jul 8;2015:473175. doi: 10.1155/2015/473175 (PMC4510257; doi:10.1155/2015/473175)
Supplement: Supplementary file 1 — The supplementary material provides additional analyses regarding the impact of HLA E on NK cell mediated cytolysis, comparative analyses of donor A and B taking into account blast HLA I genotype and NKG2DL high versus low expression on NK cytolysis. [file 473175.f1.zip › Table_S2-1389921.docx]

**Table S2. Median Fluorescence Intensity Ratio of DNAM-1 ligands, adhesion molecules, LFA-1 and 3, 2B4 and FAS Receptor**

| **AML-Blast** | **FAB** | **CD155** | **CD112** | **CD11a** | **CD18** | **CD50** | **CD54** | **CD58** | **CD48** | **CD95** |
| --- | --- | --- | --- | --- | --- | --- | --- | --- | --- | --- |
| **AML-1** | M0 | 2.29 | 14.14 | 127.94 | 11.25 | 21.54 | 4.04 | 19.25 | 1.75 | 1.65 |
| **AML-2** | M0 | 2.86 | 15.42 | 29.49 | 16.66 | 21.75 | 1.72 | 4.45 | 3.96 | 4.13 |
| **AML-3** | M2 | 3.19 | 10.78 | 173.27 | 6.90 | 19.36 | 2.20 | 8.77 | 1.12 | 1.37 |
| **AML-4** | M2 | 2.35 | 16.55 | 115.70 | 14.21 | 14.38 | 10.06 | 14.43 | 1.37 | 1.60 |
| **AML-5** | M4 | 5.3 | 14.5 | 193.45 | 53.21 | 141.18 | 0.33 | 1.04 | 3.47 | 1.98 |
| **AML-6** | M4 | 2.31 | 15.76 | 172.79 | 9.68 | 15.08 | 3.98 | 19.69 | 1.07 | 1.19 |
| **AML-7** | M5 | 2.45 | 15.40 | 52.79 | 15.81 | 8.76 | 0.41 | 2.86 | 1.04 | 1.68 |
| **AML-8** | M5 | 7.53 | 35.80 | 25.59 | 32.81 | 33.66 | 0.79 | 0.54 | 1.82 | 1.76 |
| **AML-9** | M5 | 5.05 | 20.61 | 114.26 | 35.19 | 87.01 | 0.81 | 4.24 | 2.93 | 1.37 |
| **AML-10** | M5 | 2.76 | 22.72 | 35.43 | 10.34 | 12.00 | 0.41 | 4.05 | 1.21 | 1.18 |
| **AML-11** | M5b | 3.49 | 2.96 | 26.80 | 82.67 | 47.93 | 0.95 | 0.38 | 4.28 | 2.26 |
| **AML-12** | M6 | 2.55 | 16.85 | 169.63 | 9.19 | 16.66 | 3.02 | 7.1 | 1.03 | 1.30 |
| **AML-13** | M6 | 3.75 | 3.15 | 1.31 | 1.26 | 4.80 | 2.00 | 8.02 | 1.08 | 1.05 |
| **AML-14** | M6 | 3.34 | 6.38 | 6.28 | 1.99 | 1.6 | 1.04 | 3.06 | 1.06 | 1.1 |

Median Fluorescence Intensity Ratios (MFIR) of the DNAM-1 ligands (CD155, CD112), heterodimeric LFA-1 (CD11a/CD18), LFA-3 CD58, ICAM-3 (CD50) and ICAM-1 (CD54), 2B4 (CD48) and FAS receptor (CD95) were calculated by MFI of goat anti-mouse PE divided by MFI of isotype control. To detect fluorescence, cells were incubated with unlabeled mouse anti-human ULBP1-4 antibody, then incubated with secondary PE labeled goat anti-mouse antibody and measured on a FACSCalibur^TM^ cytometer. AML-1 to AML-14 represents 14 primary childhood AML blasts. AML was classified according to the French American British Classification (FAB). MFIR < 2 was defined negative, MFIR ≥ 2 defined positive and MFIR ≥ 10 was defined highly positive.
